# Self-Medication of Medical Students at Erzincan Binali Yıldırım University in Turkey: A Cross-Sectional Study

**DOI:** 10.7759/cureus.72319

**Published:** 2024-10-24

**Authors:** Sema Turan

**Affiliations:** 1 Public Health, Erzincan Binali Yıldırım University Faculty of Medicine, Erzincan, TUR

**Keywords:** medical student, pharmacology education level, practice, self-medication, türkiye

## Abstract

Introduction: Responsible self-medication (SM) can save time, money, reduce the number of visits to the doctor, and alleviate pressure on health services. However, while there are clear benefits, SM also carries significant risks, such as increased drug side effects, inappropriate use of prescription drugs, drug interactions, misdiagnosis, drug dependence, masking of medical conditions, and antibiotic resistance.

Aim: This study aimed to investigate the practices of medical students at a public university in Türkiye towards SM, as well as to determine the factors influencing such practices.

Method: The population of this cross-sectional study included first-, fourth-, and sixth-year medical students. As the goal was to reach the entire population (375 students), no sample selection was made. A total of 332 students (88.5%) participated in the study. The relevant literature was used for the basis of the questionnaire used in the study. In the statistical evaluation, percentages, means, standard deviations, medians, minimum, and maximum values were used to present the descriptive data, while factors influencing SM were analyzed using the chi-square test and bivariate and multivariate logistic regression analysis.

Results: The overall rate of SM among the participants was found to be 96.1%. No statistically significant difference was observed in SM rates based on the year of study (p>0.05). The rate of students using antibiotics without a prescription was 19.3%. Additionally, 82.5% of the students reported reading the package insert before using a drug. The most common symptoms leading to SM were headaches (83.1%) and the common cold (63.6%). The most frequently self-administered medications were analgesics (86.1%), common cold medicines (54.8%), and vitamins (34.0%). Furthermore, 79.3% of the participants indicated that they self-medicate because they perceive their condition as a simple illness, 64.6% because they had experienced a similar illness before, and 28.2% because they believed they had sufficient knowledge of medications. In multivariate regression analysis, keeping medicines at home/in the dormitory for later use and not having a chronic disease were found to be effective factors in students' practice of SM.

Conclusion: It is important to plan educational interventions to promote the development of responsible SM behaviors. Starting these educational programs in the first year of medical school is thought to contribute significantly to the fostering of responsible SM practices

## Introduction

“Self-medication” (SM) refers to the practice of individuals selecting and using medicines independently to treat illnesses or symptoms. This behavior, which involves taking medications that are neither prescribed, recommended, nor supervised by a healthcare professional [1], can manifest in various forms, including taking medication without a prescription, using medications from an old prescription, giving medications to friends, relatives, or acquaintances, or using leftover medications [2,3].

The practice of responsible SM offers several potential advantages, including time savings, earlier access to effective treatment, reduced costs, fewer doctor visits, and alleviation of pressure on healthcare services [4]. However, while there are clear benefits, SM also increases the risk of drug side effects, alters the appropriate use of prescription medications, and can lead to interactions between non-prescription antibiotics and prescription drugs [5]. Furthermore, SM can result in drug dependence, toxicity, misdiagnosis, the masking of medical conditions, antibiotic resistance, increased healthcare costs, birth defects, maternal mortality, psychological symptoms during pregnancy, and addiction [6,7]. Mild symptoms are a primary driver of SM, a common practice that affects individuals worldwide, with respiratory diseases, colds, and headaches being among the most common conditions leading to SM. The most frequently self-administered medications include analgesics, cough and common cold medicines, antibiotics, antacids, and vitamins [8,9].

SM is prevalent worldwide. A meta-analysis conducted in Ethiopia reported an SM prevalence of 49.4% among university students [9]. Furthermore, a systematic review conducted in low- and middle-income countries found a 57.8% prevalence of SM among primary healthcare workers, with antibiotic use at 61.5% [10]. In a meta-analysis from India, the prevalence of SM was 53.6% [11]. Additionally, a meta-analysis which examined the prevalence of SM and its associated factors in pregnant women reported a prevalence of 44.5% [12]. According to a WHO regional and continental meta-analysis on self-medication, the highest incidence of self-medication globally is in Eastern European and Asian countries [13]. Studies focusing on medical students reveal that they are more likely to self-medicate compared to the general population [14-16].

As with other countries, SM is also common in Türkiye. Studies conducted in various groups show that the prevalence of SM in Türkiye ranges from 26.3% to 89.9% [17-21]. However, research specifically evaluating SM among medical students in Türkiye remains limited.

The aim of this study was to assess the SM practices of medical students with different levels of pharmacology education, while also identifying the factors which influenced SM use at Erzincan Binali Yıldırım University Faculty of Medicine.

## Materials and methods

Study design and setting

This cross-sectional study was conducted at a public university medical faculty in Erzincan province in eastern Turkey between July and September 2022. The study design involved the completion of questionnaires by medical students.

Sample size calculation and sampling technique

The population of the study consisted of Erzincan Binali Yıldırım University Faculty of Medicine first, fourth and sixth grade students. Since it was planned to reach the whole population (375 students), no sample was selected. One hundred fifty-eight of 160 first-year students (98.8%), 115 of 140 fourth-year students (82.0%) and 59 of 75 sixth-year students (78.7%) participated in the study. The total number of students participating in the study was 332 (88.5%).

The study was conducted on first-grade students who had never received pharmacology education, fourth-grade students who received theoretical pharmacology education and sixth-grade students who received both theoretical and clinical pharmacology education. At the medical faculty where this study was conducted, basic education in pharmacology is provided in the second and third years, while clinical pharmacology is taught in the fifth year. Students complete their theoretical and clinical education in the first five years and graduate after fulfilling their compulsory and elective internships during the sixth year.

The inclusion criteria were students who voluntarily agreed to participate in the study and gave informed consent. The exclusion criteria were students who did not want to participate in the study and were not present at the school on the day the questionnaire was administered. By using convenience sampling method, it was tried to reach the students once for the questionnaire application. 

Data collection and the study tool

Questionnaire technique under observation was used to collect data in the study. The questionnaire used in the study consists of 23 questions prepared by the researcher by utilising the literature on the subject. The questionnaire was first applied to a group of 15 people. The questionnaire was reviewed for clarity and correction of errors and finalised by making the necessary adjustments. The questionnaire consists of three parts. In the first part of the questionnaire form, the participants' sociodemographic information such as age, gender, residence information, high school education, parental education and personal habits such as chronic diseases, smoking and exercise, in the second part, self-medication use, reasons for self-medication use, symptoms that cause the most self-medication use, the most self-medicated drugs, the results of self-medication use and self-medication practices such as keeping medicines at home/dormitory. Exercise was assessed as those who regularly exercised one hour or more per week and those who regularly exercised less than one hour per week. In the third part, there are questions evaluating the status of reading/understanding the drug package insert and knowing the term rational use of medicine (RUM).

After the information was provided by the researcher, the questionnaires were distributed to the participants and the completed questionnaires were collected. During the completion of the questionnaires, the questions of the participants were answered by the researcher. The first-grade students were reached in the lecture halls where they did theoretical courses, and the fourth- and sixth-grade students were reached in the clinics where they did internships.

Statistical analysis

The data of the study were analysed with the statistical package programme SPSS 22.0 (IBM Corp., Armonk, NY, USA). In the statistical evaluation, percentage, mean, standard deviation, median, median, minimum, maximum values were used in the presentation of descriptive data. Factors affecting self-medication use were analysed by chi-square test with p value <0.05 was considered statistically significant. Finally, bivariate and multivariate logistic regression analysis were employed to identify variables linked with self-medication practices. Bivariate analysis was done and variables with a p value <0.25 were further taken to the multivariate logistic regression analysis for proper adjustment with the possible confounders. In our study, adjusted odds ratio at 95% confidence interval (95% CI) with p value <0.05 was considered statistically significant.

Ethical considerations

The research was approved by Erzincan Binali Yıldırım University Human Research Health and Sports Ethics Committee (Date: 29.06.2022, Protocol No: 06/06). Students were informed by the researcher about the aims of the study and informed consent was obtained from the students who voluntarily agreed to participate in the study. All data were securely stored by the researcher and used only for the purpose of the study.

## Results

The mean age of the 332 students who participated in the study was 21.2±2.5 years, with a median of 21 (Min: 17, Max: 42). Of the participants, 52.7% were female, and 47.6% were first-year students. It was found that 20.5% of the students smoked and 38.9% exercised regularly. The rate of SM among all students was 96.1%, with no statistical difference in SM rates across different years of education (p>0.05). However, students who kept medicines at home, or in their dormitories for later use, had a significantly higher rate of SM (p<0.001). The self-medication status of the students according to their descriptive characteristics is presented in Table 1.

**Table 1:** SM Status of Students According to Descriptive Characteristics (n=332) *Column percentage, **Row percentage,  ^β^ Fisher’s Exact test, all without the (^β^) symbol are Pearson Chi-Square tests, Significant value p<0.05, SM= Self Medication

Variables	n(%)*	SM n(%)**		
		Yes	No	Test
Gender				
Female	175 (52.7)	170 (97.1)	5 (2.9)	X^2=^1.102 (df=1) p=0.294
Male	157 (47.3)	149 (94.9)	8 (5.1)	
Type of High School Education				
Daytime	262 (78.9)	254 (96.9)	8 (3.1)	p=0.158^ β^
Boarding	70 (21.1)	65 (92.9)	5 (7.1)	
Grade				
1st-year	158 (47.6)	153 (96.8)	5 (3.2)	p=0.704^ β^
4th-year	115 (34.6)	109 (94.8)	6 (5.2)	
6th-year	59 (17.8)	57 (96.6)	2 (3.4)	
Residence				
with Family	43 (13.0)	43 (100.0)	0	p=0.388^ β^
in Dormitory	289 (87.0)	276 (95.5)	13 (4.5)	
Mother’s Education				
up to High School	161 (48.5)	155 (96.3)	6 (3.7)	X^2 ^=0.030 (df=1) p=0.863
High School and above	171 (51.5)	164 (95.9)	7 (4.1)	
Father’s Education				
up to High School	80 (24.1)	78 (97.5)	2 (2.5)	p=0.741^ β^
High School and above	252 (75.9)	241 (95.6)	11 (4.4)	
Chronic Illness				
Yes	35 (10.5)	32 (91.4)	3 (8.6)	p=0.147^β^
No	297 (89.5)	287 (96.6)	10 (3.4)	
Smoker				
Yes	68 (20.5)	65 (95.6)	3 (4.4)	p=0.734^β^
No	264 (79.5)	254 (96.2)	10 (3.8)	
Regular Exercise				
Less than 1 hour per week	129 (38.9)	123 (95.3)	6 (4.7)	X^2 ^= 0.303 (df=1) p=0.582
1 hour or more per week	203 (61.1)	196 (96.6)	7 (3.4)	
Keeps Medicine at Home/Dormitory for Later Use				
Yes	235 (70.8)	233 (99.1)	2 (0.9)	p<0.001^β^
No	97 (29.2)	86 (88.7)	11 (11.3)	
Reading the Drug Package Insert				
Yes	274 (82.5)	263 (96.0)	11 (4.0)	p=1.000^ β^
No	58 (17.5)	56 (96.6)	2 (3.4)	
Understanding the Drug Package Insert				
Yes	186 (56.0)	177 (95.2)	9 (4.8)	X^2 ^=0.958 (df=1) p=0.328
No/Partially	146 (44.0)	142 (97.3)	4 (2.7)	
Awareness of the Term ‘Rational Drug Use’				
Yes	205 (61.7)	198 (96.6)	7 (3.4)	X^2 ^=0.358 (df=1) p=0.550
No	127 (38.3)	121 (95.3)	6 (4.7)	

The rate of students using antibiotics without a prescription was 19.3%. This rate was 22.8% in the first grade, 10.4% in the fourth grade, and 27.0% in the sixth grade. The difference in non-prescription antibiotic use rates according to grade levels was statistically significant (X² (df=2)=9.359, p=0.09). This statistical difference is attributed to the lower rate of using antibiotics without a prescription in the fourth grade compared to both the first and sixth grades. The rate of students who discontinued antibiotics inappropriately (before the time recommended by the doctor or without consulting the doctor) was 28.6%. The proportion of students reporting inappropriate antibiotic discontinuation was 34.2% in the first grade, 22.6% in the fourth grade, and 25.4% in the sixth grade, with no statistical difference between the grades (X² (df=2)=4.718, p=0.095).

The rate of using antibiotics without a prescription was 86.5% among students who inappropriately discontinued antibiotics, compared to 66.3% in those who used antibiotics appropriately, with the difference between both groups being statistically significant (X² (df=1)=17.751, p<0.001).

The rate of students familiar with the term RUM was 50.6% in the first grade, 63.5% in the fourth grade, and 88.1% in the sixth grade, and the difference between the classes was statistically significant (X² (df=2)=25.803, p<0.001). This statistical difference is due to the higher rate of familiarity with the term RUM among sixth graders compared to both fourth and first graders.

When the medicines students kept at home or in the dormitory for later use were examined, the most common were common cold medicines (82.1%), analgesics (54.0%), vitamins (43.4%), antibiotics (31.1%), antihistamines (18.7%), and antacids (12.8%). During SM or after self-treatment, 0.6% of the students stated that they experienced side effects due to the medication they used, 13.5% rarely experienced side effects and 85.9% did not experience side effects. The rate of students reporting complete recovery as a result of SM was 85.6%. 11.6% of the students reported rare complete recovery, and 2.8% reported incomplete recovery.

The leading symptoms that led to SM were headache (79.8%), common cold (61.1%) and fever (38.6%) (Table 2).

**Table 2:** Symptoms Causing SM in Students *Students selected more than one option, SM = Self Medication

Symptoms	n*	Percentage (%)
Headache	265	79.8
Common cold	203	61.1
Fever	128	38.6
Stomach pain/reflux	113	34.0
Coughing	86	25.9
Seasonal allergy	57	17.2
Muscle/joint pain	42	12.7
Constipation/diarrhea	32	9.6
Skin complaints	30	9.0
Eye complaints	26	7.8
Dysmenorrhea	15	4.7
Other (toothache, weakness/fatigue)	2	0.6

The most frequently self-administered medicines were analgesics (86.1%), common cold medicines (54.8%), and vitamins (34.0%) (Table 3). When the reasons for SM were analyzed, 76.2% of the participants stated that it was due to a simple illness, 62.0% reported having had a similar illness before, and 27.1% believed they had sufficient knowledge of medications (Table 4).

**Table 3:** Most Commonly Self-administered Medicines * Students selected more than one option

Most commonly used medicines	n*	Percentage (%)
Analgesics	286	86.1
Common cold medicines	182	54.8
Vitamins	113	34.0
Stomach medications (antacids, proton pump inhibitors)	64	19.3
Topical skin medicines	57	17.2
Antitussives, mucolytics	52	15.7
Bowel regulators	38	11.4
Antibiotics	32	9.6
Nasal sprays	31	9.3
Eye drops	26	7.8
Antihistamines	17	5.1

**Table 4:** Reasons Leading to SM in Students *Students selected more than one option, SM = Self Medication

Reasons leading to SM	n*	Percentage (%)
Having a simple illness	253	76.2
Previous history of similar illness	206	62.0
Believing they have sufficient knowledge of medications	90	27.1
To save time	62	18.7
Due to urgent symptoms	28	8.4
Difficulty in reaching a doctor	20	6.0
To save money	8	2.4
Friend’s recommendation	7	2.2
Ineffectiveness of the prescribed medicine	6	1.8
Other (patient density in the hospital, lack of social security)	3	0,9

Variables substantially correlated with self-medication habits in the bivariate study were subsequently analyzed using multivariate logistic regression. In binary logistic regression analyses, daytime high school education, not having a chronic disease and keeping medication at home/in the dormitory for later use were associated with SM at p-value < 0.25. In multivariate logistic regression analyses, not having a chronic disease and keeping medication at home/in the dormitory for later use were significantly associated with SM at the p-value < 0.05 level. The likelihood of practising SM was nearly five times higher in those without chronic disease than in those with chronic disease (AOR=5.14 95%CI=1.12-23.64 p=0.035). Those who kept medication at home/dormitory for later use were nearly 18 times more likely to practice SM than those who did not keep medication at home/housing for later use (AOR=17.62, 95%CI=3.64-85.17, p<0.001) (Table 5).

**Table 5:** Factors Associated with SM Practices Among Students (n=332) *Significant value p<0.25  ** Significant value p<0.05 SM=self medication, OR=odds ratio, CI=confidence interval, ref.=reference

Variables	Crude OR (95% CI)	p*	Adjusted OR (95% CI)	p**
Type of High School Education (ref. Boarding)				
Daytime	2.44 (0.77-7.71)	0.128	2.96 (0.84-10.41)	0.091
Chronic Illness (ref. Yes)				
No	2.69 (0.70-10.29)	0.148	5.14 (1.12-23.64)	0.035
Keeps Medicine at Home/Dormitory for Later Use (ref. No)				
Yes	14.90 (3.24-68.60)	0.001	17.62 (3.64-85.17)	<0.001

## Discussion

In this study, practices towards self-medication (SM) were evaluated among first-, fourth-, and sixth-year medical students, each with different levels of pharmacology education, at a public university medical faculty in Erzincan, Türkiye.

The level of self-medication was found to be very high at 96.1%. In studies conducted among medical students in Türkiye, this rate has been shown to vary between 41.9% and 83.1% [22-26]. Similarly, the high prevalence of SM among medical students has been demonstrated in studies from other countries. For instance, the rate of SM was found to be 93.0% in a study by Awori and Onyango in Uganda [27], 81.3% in a study by Ramadan in Iraq [28], and 83.8% in a study by Sajjad et al. in Pakistan [29]. Medical students receive comprehensive training in basic and clinical pharmacology throughout their education, which may contribute to higher levels of SM. However, as a group, they are also expected to be more knowledgeable about the risks associated with SM.

In our study, no relationship was found between the year of education and the level of SM. Similarly, studies conducted by Kartal et al. in Türkiye [26] and Mirdoosti et al. in Iran [30] on different grades of medical students found no significant difference in SM levels based on the year of education. Likewise, a study conducted in Iraq investigating self-administration of analgesics among medical students found no significant relationship between students’ academic level and analgesic use [31]. However, some studies in the literature have shown that the level of SM increases with advancing years of medical education. For example, research conducted in India [32], Pakistan [29], and Serbia [33,34] demonstrated that SM levels rose as students progressed through their medical training. These studies indicate that the elevated prevalence of SM among students at advanced academic levels is due to the increased knowledge of pharmacology as medical students approach their final year, which subsequently enhances their confidence in diagnosing and selecting appropriate treatments for diseases.

According to our results, keeping medicines at home or in the dormitory for later use significantly increased the likelihood of SM. This finding is consistent with studies by Niroomand et al. in Iran [35], Lukovic et al. in Serbia [34], and Helal and Abou-ElWafa in Egypt [15], which also reported that having medication stock at home was associated with a higher likelihood of SM. In our study, participants without chronic diseases were more likely to self-medicate. This suggests that students with chronic diseases are more cautious due to risks such as interaction with their current medications or possible side effects and that they resort to SM practice less frequently. On the other hand, in the studies of Kartal et al. and Petrovic et al. no relationship was found between having a chronic disease and the practice of self-medication [26,33].

According to our findings, there was no difference between SM practice according to gender, residence, chronic disease, smoking and regular exercise. Similarly, in a study conducted in health sciences students in Iran, no difference was found according to gender and place of residence [36]. Again, in a study conducted in Ethiopia in medical and non-medical university students, no difference was found in SM practice between genders [37]. Differently, in a study conducted in university students in Turkey, the prevalence of SM was found to be higher in females [38] and in males in a study conducted in medical and pharmacology students in Iran [39]. In a study conducted in medical students in Serbia, low educational level of the father was found to be a factor increasing the likelihood of SM and this was attributed to the effect of family attitudes. In the same study, students who engaged in less than one hour of physical activity per week were found to self-medicate more than other students [34]. Another study conducted among medical and pharmacology students in Serbia showed that students living independently from their parents in a rented apartment or student dormitory had a significantly higher prevalence of SM compared to those living with their parents. In the same study, SM levels were found to be higher in smokers compared to non-smokers [33].

The rate of using antibiotics without a prescription (19%) among the students who participated in our study is similar to those in other studies conducted on medical students in Türkiye [22,26]. The self-administration of antibiotics at different levels has also been identified in studies conducted on medical students in other countries. This rate was found to be 25% in Saudi Arabia [4], 27% in China [40], and 60.8% in Sudan [41].

The fact that the use of non-prescription antibiotics was significantly lower among fourth-year students suggests that basic pharmacology education has a positive impact. However, the increase in this rate among sixth-year students indicates that increasing knowledge of Clinical Pharmacology and other clinical training may contribute to this rise. As the sale of antibiotics without a prescription is prohibited in pharmacies in Türkiye, it is likely that self-administered antibiotics are left over from previous treatments that were not fully completed. In fact, the rate of non-prescription antibiotic use was found to be significantly higher in those who had erroneously not completed their course of antibiotics.

According to our results, it is noteworthy that one-fourth of the sixth-year students who had completed both their theoretical and clinical education still left their antibiotics unfinished. This finding demonstrates that some sixth-year students were unable to practice what they had learned. In a study conducted by Kukula on medical students in Türkiye, 38.7% reported that they discontinued antibiotic use once their symptoms subsided [42]. This is despite one of the most common causes of antibiotic resistance in microorganisms being the inappropriate use of antibiotics, including prematurely discontinuing treatment [43].

Our study showed that headache, common cold, fever, and stomach complaints were the most common symptoms that led to SM among students. The most frequently self-administered medications were analgesics, common cold medicines, vitamins, and stomach medications. In studies conducted both in Türkiye and other countries, the symptoms causing SM, and the types of medications used, are similar [4,9,18,19,26,28,44]. Analgesics (painkillers), which are the most commonly used medications, are easily accessible without a prescription and are cheap in Türkiye.

The main reasons cited by participants for SM were as follows: it was a simple illness, a similar illness had been experienced before, and participants believed they had sufficient knowledge of medications. Similarly, in a study conducted among medical and pharmacy students in Saudi Arabia, mild health issues and prior experiences with medications [45], and in a study among medical students in Nepal, knowledge of medications and medical conditions [46], were identified as key reasons for self-medication. Likewise, in India, most medical students self-administered medications because they believed the illness to be mild [32]. It can be concluded that as many diseases share similar symptoms, high self-confidence and positive attitude toward self-medication among medical students is likely to lead to an increased risk of misdiagnosis and inappropriate treatment.

In our study, it was encouraging to find that the majority of students (82.5%) reported reading the package insert of the drugs they use, as this is a positive behavior considering the risks of side effects and drug interactions associated with SM. This rate was similar to that observed in a study conducted among pharmacy students in Jordan (83.2%) [47]. The significantly higher rate of awareness of the term RUM among sixth-year students, compared to other years, suggests that study of Clinical Pharmacology has a positive effect. As physicians play a crucial role in promoting RUM within society, it is clearly essential for medical students to understand the principles of RUM and adopt appropriate behaviors.

There are certain limitations with the study. As it was conducted at a single medical faculty, the results cannot be generalized for all medical students in the country. Another limitation is that the study is based on self-reported data, which may lead to inaccuracies due to forgetfulness or recall bias. Multicentre studies and qualitative research on the subject may provide more information on understanding the causes of SM among medical students and solutions to reduce it.

## Conclusions

In our study, the rate of SM was found to be very high among medical students and the year of education did not significantly affect the level of SM. However, keeping medications at home or in dormitories for later use was identified as a key factor in increasing SM levels. In addition, not having a chronic disease has been found to be a factor that increases SM implementation. Headache, common cold, fever, and stomach complaints were found to be the most common symptoms leading to SM, with analgesics, common cold medicines, vitamins, and stomach medications being the most frequently self-administered drugs. Although the high percentage of sixth-year students who were familiar with the term RUM reflects the positive impact of education in Clinical Pharmacology education, it is concerning that one-fourth of these students left their antibiotics unfinished, and this highlights the gap between knowledge and behavior.

In conclusion, it is recommended that educational programmes promoting responsible SM behaviour should be designed for medical students and SM education should be included from the first year onwards. Emphasising the negative consequences of inappropriate self-medication through such programmes and trainings is thought to encourage more responsible attitudes and practices among students.
